# Antiviral activity of pimecrolimus against dengue virus type 2 infection in vitro and in vivo

**DOI:** 10.1038/s41598-024-61127-x

**Published:** 2024-06-10

**Authors:** Seong-Ryeol Kim, Jung-Min Lee, Hae Ji Kang, Jungsang Ryou, Sang-Mu Shim

**Affiliations:** Division of Acute Viral Diseases, Centers for Emerging Virus Research, National Institute of Infectious Disease, Korea National Institute of Health, Korea Disease Control and Prevention Agency, Cheongju-si, Chungcheongbuk-do Republic of Korea

**Keywords:** Dengue virus, Drug development

## Abstract

Dengue virus (DENV) infection is a public health concern in several countries and is associated with severe diseases, such as dengue hemorrhagic fever and dengue shock syndrome. DENVs are transmitted to humans via the bites of infected *Aedes* mosquitoes, and no antiviral therapeutics are currently available. In this work, we aimed to identify antiviral drugs against DENV type 2 (DENV2) infections and selected pimecrolimus as a potential antiviral drug candidate. Pimecrolimus significantly inhibited DENV2-mediated cell death and replication in vitro. We also confirmed a decrease in the number of plaques formed as well as in the envelope protein levels of DENV2. The time-of-addition and course experiments revealed that pimecrolimus inhibited DENV2 infection during the early stages of the virus replication cycle. In an experimental mouse model, orally administered pimecrolimus alleviated body weight loss and lethality caused by DENV2 infection, which we used as readouts of the drug’s antiviral potency. Furthermore, pimecrolimus significantly inhibited the DENV2 load and ameliorated focal necrosis in the liver and spleen. Taken together, our in vitro and in vivo findings suggest that pimecrolimus is a promising antiviral drug candidate for the treatment of DENV2 infection.

## Introduction

Dengue virus (DENV) is an enveloped virus containing an ~ 11 kb genome of a single positive-strand RNA, which encodes three structural proteins (capsid [C], envelope [E], and membrane [M]) and seven non-structural proteins (NS1, NS2A, NS2B, NS3, NS4A, NS4B, and NS5). DENV is a member of the flavivirus family *Flaviviridae* and is transmitted by *Aedes aegypti* and *Aedes albopictus* mosquitoes, thereby making it an important mosquito-borne disease virus, similar to the Zika virus, West Nile virus, and Japanese encephalitis virus^1^. There are four DENV serotypes: DENV1, DENV2, DENV3, and DENV4^2^.

An estimated 390 million cases of dengue infection are reported globally, of which 96 million show mild-to-severe clinical manifestations^3^, with approximately 25,000 deaths recorded annually^4^. DENV infection can cause acute symptoms including high fever, headaches, and joint pain^5^. Recently, several antiviral candidates for DENV, including chloroquine, celgosivir, lovastatin, balapiravir, and prednisolone, have been examined; however, they have failed to meet the efficacy endpoints^6^.

Serine/threonine phosphatase calcineurin inhibitors, including pimecrolimus, ascomycin, and tacrolimus, bind to the immunophilin macrophilin-12^7^. Pimecrolimus is an immunomodulatory macrolactam commonly used to treat inflammatory skin diseases, and its mechanism of action involves the inhibition of the host immune response via T helper cells, mast cells, and basophils^8^. Pimecrolimus and tacrolimus have been reported to regulate cellular functions, such as stress and antifungal responses^9,10^. Ascomycin has also been reported to contribute to biological activities, such as immunosuppression^11,12^, as well as antifungal ^12^ and antiviral^1^ responses. However, the specific antiviral activity of pimecrolimus against DENV is unknown.

We screened 1,971 FDA-approved drugs and found pimecrolimus as a possible drug candidate against DENV2. In this study, we demonstrated the antiviral activity of serine/threonine phosphatase calcineurin inhibitors, including ascomycin and tacrolimus, which have similar structures to that of pimecrolimus. Furthermore, we assessed the antiviral activity of pimecrolimus in vivo, as it exhibited the highest antiviral activity against DENV2 in vitro among the tested compounds.

## Results

### Pimecrolimus treatment reduces intracellular DENV2 production

**Figure 1:**
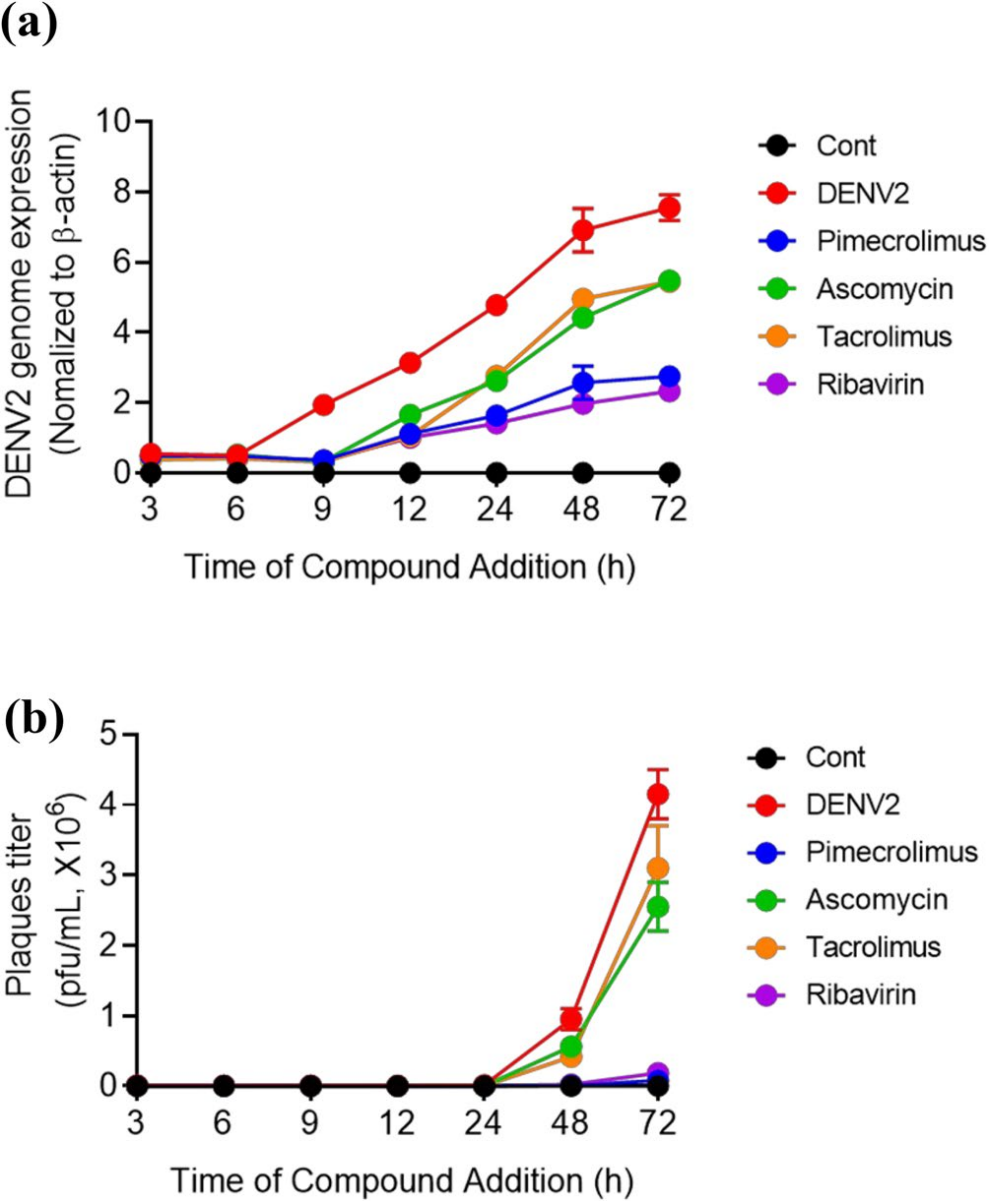
Inhibition of dengue virus type 2 (DENV2) production by pimecrolimus, ascomycin, and tacrolimus in Vero cells,Vero cells were infected with DENV2 at 0.1 multiplicity of infection (MOI) and treated with 20 μΜ pimecrolimus, ascomycin, or tacrolimus and 500 μΜ ribavirin. (**a**) Relative DENV2 genome expression across the various treatments was determined via real-time PCR at different time points. (**b**) Culture supernatants at different time points were used to infect BHK-21 cells. DENV2 titers were determined using plaque assays. Data are expressed as mean ± SEM (n = 3 independent biological experiments, each conducted with triplicate technical repeats).

To investigate the antiviral effects of 1,2-disubstituted piperidines, including pimecrolimus, ascomycin, and tacrolimus, we first designed viral genome expression and plaque formation assays to detect their inhibitory effects against DENV2 infection in Vero cells. The expression of the DENV2 genome was detected 9 h after infection; however, in the pimecrolimus-, ascomycin-, tacrolimus-, or ribavirin-treated cell cultures, the earliest time point when viral RNA expression was detected was 12 h after infection. In addition, pimecrolimus treatment resulted in lower DENV2 genome expression than treatment with ascomycin or tacrolimus (Fig. 1a). Pimecrolimus reduced DENV2 production more effectively compared to the DENV2 production observed in the other treatment groups 48 h post-infection (hpi) (Fig. 1b). Ribavirin has been previously reported to exhibit anti- DENV2 activity and has been used as a reference drug^13,14^. Our results indicated that pimecrolimus could reduce the production of infectious DENV2 particles. We also evaluated the cytotoxic effects of pimecrolimus, ascomycin, tacrolimus, and ribavirin in Vero and BHK-21 cells using the cell viability assay, and the corresponding cytotoxic concentration (CC_50_) and inhibitory concentration (IC_50_) values are presented in Table 1. Pimecrolimus, ascomycin, and tacrolimus were not cytotoxic, and pimecrolimus presented with lower IC_50_ values than those of ascomycin and tacrolimus in Vero and BHK-21 cells (Table 1).

**Table 1:** Anti-DENV2 efficacy of pimecrolimus, ascomycin, and tacrolimus.

Compounds	Vero		BHK-21	
	CC_50_^a^	IC_50_^b^	CC_50_^a^	IC_50_^b^
Pimecrolimus	> 20	3.4 ± 0.1	> 20	3.2 ± 0.1
Ascomycin	> 20	16.5 ± 0.1	> 20	14.6 ± 0.7
Tacrolimus	> 20	14 ± 0.2	> 20	11.6 ± 0.3
Ribavirin	> 500	122.8 ± 6.6	> 500	68.4 ± 3.8

^a^Concentration required to reduce cell growth by 50% (μM).

^b^Concentration required to inhibit virus-induced cytopathic effect by 50% (μM).

### Pimecrolimus acts at the viral entry stage during DENV2 infection

**Figure 2:**
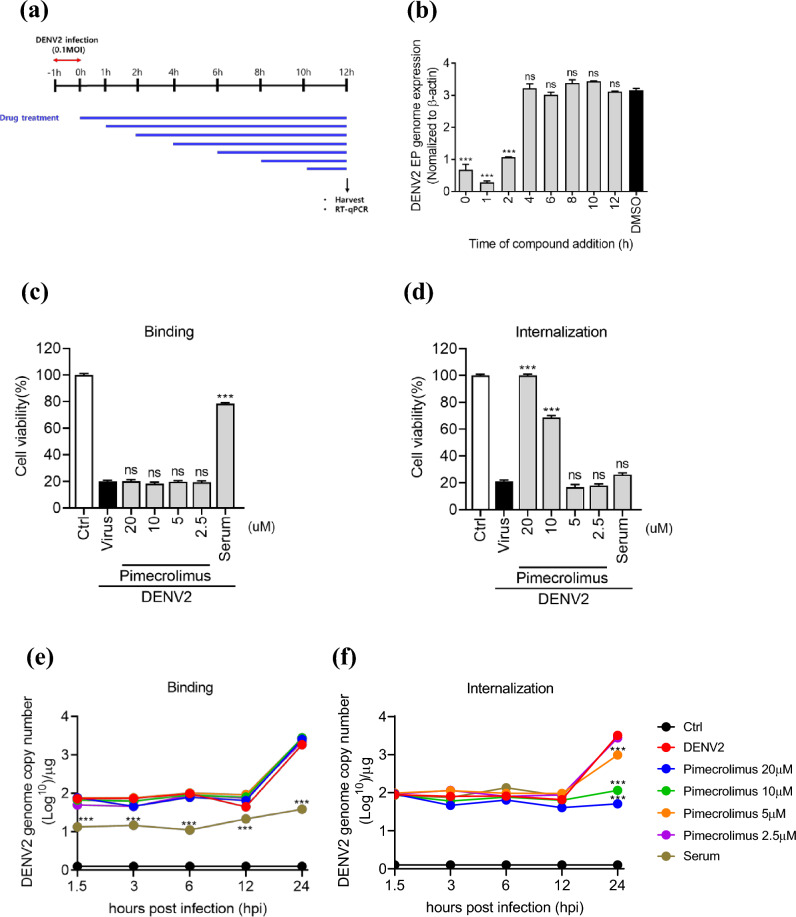
Pimecrolimus inhibits DENV2 replication at an early stage of infection. (**a**) Experimental scheme of the time-of-addition assay. (**b**) Vero cells infected with 0.1 MOI of DENV2. Pimecrolimus at 20 μΜ was added at the indicated time points, and genome expression was analyzed 12 h post-infection using real-time PCR. **(c**) Binding inhibition assay and (**d**) internalization inhibition assay of 0.1 MOI DENV2-infected Vero cells treated with the indicated concentrations of pimecrolimus at 96 hpi using cell viability assay. (**e**) Binding inhibition assay and (**f**) internalization inhibition assay of 0.1 MOI DENV2-infected Vero cells treated with the indicated concentrations of pimecrolimus at different time points via real-time PCR for DENV2 genome copy number. Data are expressed as mean ± SEM (n = 3 independent biological experiments, each conducted with triplicate technical repeats; ns, not significant; ***p < 0.001; comparisons were made against the DENV2-infected and non-infected control groups.

To clarify which step of the viral life cycle is affected by pimecrolimus, we conducted a time-of-addition assay. Vero cells were infected with DENV2 at a 0.1 multiplicity of infection (MOI) for 1 h, and the culture medium was replaced with medium containing 20 μM pimecrolimus at 0, 1, 2, 4, 8, 10, and 12 hpi (Fig. 2a). Culture supernatants were collected for real-time PCR at 12 hpi. We observed that when pimecrolimus was added at the early stage (0–2 h), there was a significant inhibition of DENV2 infectivity compared to that in the non-infected control cells; however, when pimecrolimus was added at the late stage (4–12 h), there was no difference in DENV2 infection relative to that in the control group (Fig. 2b). Next, we evaluated whether pimecrolimus affects the early stages of DENV2 infection in host cells. On the basis of the results of viral binding and internalization assays, pimecrolimus did not affect viral binding (Fig. 2c,e). Notably, we observed that treatment with 10–20 μM pimecrolimus significantly reduced the internalization of DENV2 at 96 hpi (Fig. 2d); similarly, Vero cells treated with 5–20 μM pimecrolimus showed significantly reduced genome copy number of DENV2 at 24 h (Fig. 2f). Collectively, these results suggest that pimecrolimus inhibits viral internalization during DENV2 infection.

### Pimecrolimus exhibits antiviral activity against DENV2 and Zika virus (ZIKV) infection in vitro

**Figure 3:**
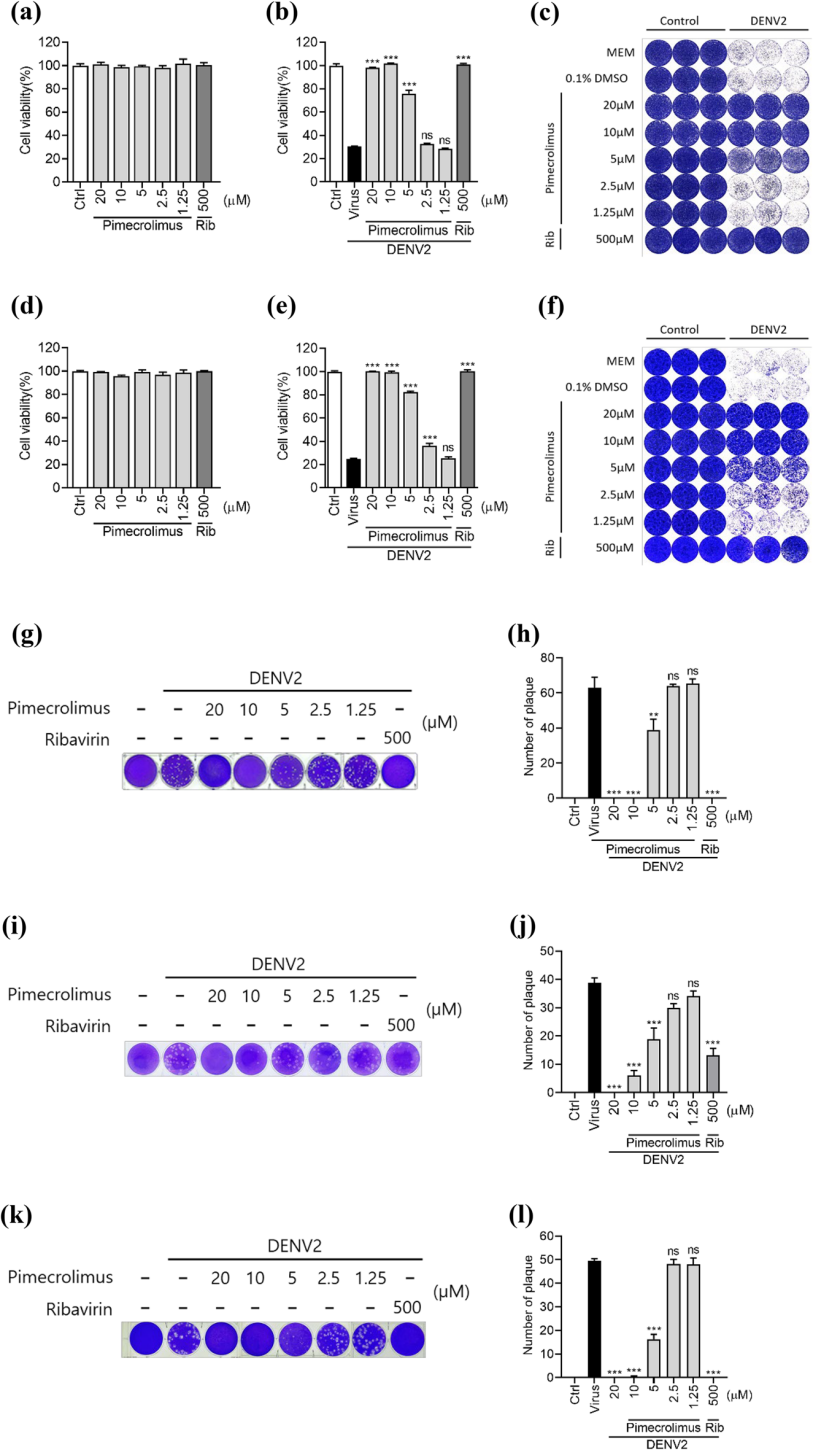
Pimecrolimus exhibits antiviral activity against DENV2 infection, (**a**) Viability of Vero cells treated with the indicated concentrations of pimecrolimus or ribavirin without virus for 4 dpi. (**b**) Vero cells infected with 0.1 MOI of DENV2 and treated with the indicated concentrations of pimecrolimus and ribavirin for 4 dpi. (**c**) Representative images of the crystal violet staining for visually comparing the viability of control and DENV2-infected Vero cells across the different compound concentrations used. (**d**) BHK-21 cells treated with the indicated concentrations of pimecrolimus and ribavirin without virus for 2 dpi. (**e**) BHK-21 cells infected with 0.1 MOI of DENV2 and treated with the indicated concentrations of pimecrolimus and ribavirin for 2 dpi. (**f**) Representative images of the crystal violet staining for visually comparing the viability of control and DENV2-infected BHK-21 cells across the different compound concentrations used. (**g**) Plaque reduction assay in DENV2-infected BHK-21 cells treated with the indicated concentrations of pimecrolimus and ribavirin. After 4 days, cells were stained with 0.2% crystal violet, and representative images of cell viability results are shown. (**h**) Comparison of the relative number of plaques formed in BHK-21 cells across the different pimecrolimus or ribavirin treatment concentrations used. (**i**) Plaque reduction assay in DENV2-infected Vero E6 cells treated with the indicated concentrations of pimecrolimus and ribavirin. After 7 days, cells were stained with 0.2% crystal violet, and representative images of cell viability results are shown. (**j**) Comparison of the relative number of plaques formed in Vero E6 cells across the different pimecrolimus or ribavirin treatment concentrations used. (**k**) Plaque reduction assay on DENV2-infected Huh-7 cells treated with the indicated concentrations of pimecrolimus and ribavirin. After 7 days, cells were stained with 0.2% crystal violet. Representative images of cell viability results are shown. (**l**) Comparison of the relative number of plaques formed in Huh-7 cells treated with different pimecrolimus and ribavirin concentrations. Data are expressed as mean ± SEM (n = 3 independent biological experiments, each conducted with triplicate technical repeats; ns, not significant; ***p < 0.001; comparisons were made against the DENV2-infected group).

To confirm the antiviral activity, DENV2-infected Vero and BHK-21 cells were incubated with the indicated concentrations of pimecrolimus, and cell viability was evaluated using the WST-1 assay. Pimecrolimus was added to 0.1 MOI of DENV2-infected cells by two-fold serial dilutions, starting with a concentration of 20 μΜ. Pimecrolimus and ribavirin were not cytotoxic at any concentration in Vero (Fig. 3a) or BHK-21 cells (Fig. 3d). The DENV2 infection resulted in approximately 70% cell death in Vero E6 and BHK-21 cell cultures; however, treatment with 5 μM pimecrolimus significantly increased cell viability, which ranged from 75 to 80%, with 10–20 μM pimecrolimus completely abolishing any virus-induced cytopathic effect (Fig. 3b,e). In addition, the number of DENV2-infected Vero E6 and BHK-21 cells decreased following pimecrolimus treatment in a dose-dependent manner (Fig. 3c,f). The DENV2-induced plaque formation in BHK-21, Vero E6, and Huh-7 cells also decreased with increasing pimecrolimus concentrations (Fig. 3g–l).

The previously reported analog compounds, ascomycin and tacrolimus, were compared for their antiviral effects against DENV2 and ZIKV infections. The viability of DENV2-infected Vero E6 and BHK-21 cells treated with pimecrolimus was significantly higher than that of cells treated with ascomycin alone (Supplementary Fig. S1) or tacrolimus (Supplementary Fig. S1). Cell viability assays indicated no cytotoxicity at concentrations of 1.25–20 μM for ascomycin (Supplementary Fig. S1) and tacrolimus (Supplementary Fig. S1). As shown in Fig. 1g,h, pimecrolimus treatment significantly reduced DENV2-mediated plaque formation in Vero E6 cells. ZIKV-mediated plaque formation also decreased following treatment with 20 μM pimecrolimus in Vero E6 cells (Supplementary Fig. S2). However, treatment with ascomycin (Supplementary Fig. S2) and tacrolimus (Supplementary Fig. S2) did not have any effect on ZIKV infection. These findings indicate that pimecrolimus also exerts antiviral effects against ZIKV.

### Pimecrolimus treatment reduces intracellular DENV2 viral protein production

**Figure 4:**
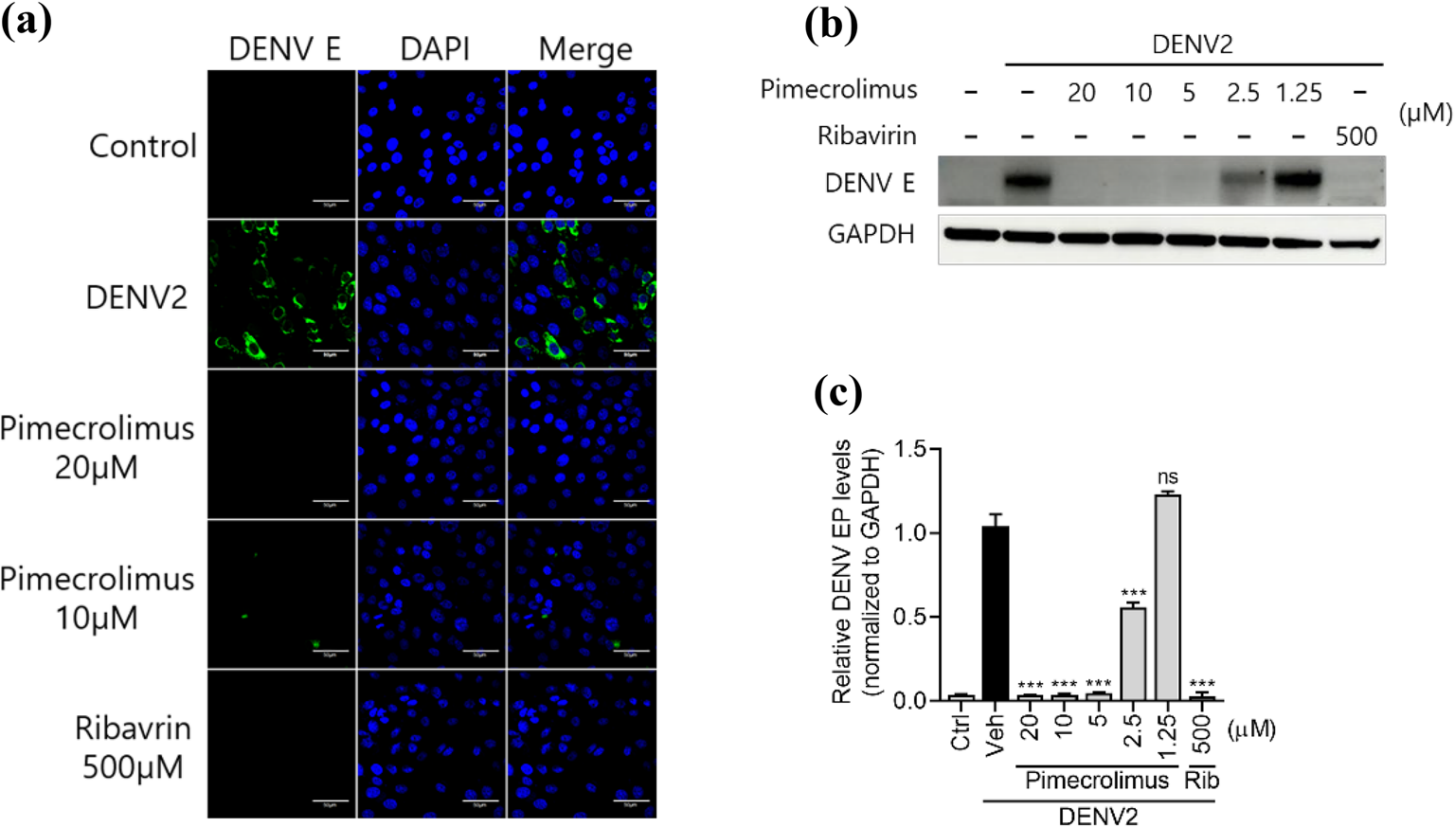
Pimecrolimus limits DENV2 localization in Vero cells, (**a**) Immunofluorescence images of Vero cells infected with 0.1 MOI of DENV2 and treated with pimecrolimus or ribavirin at the indicated concentrations for 48 h. The expression of the DENV envelope (DENV E) protein was used to distinguish DENV2-positive cells. Nuclei are stained with DAPI. Scale bar: 50 μm. (**b**) Huh-7 cells infected with 0.1 MOI of DENV2 for 72 h at the indicated concentrations of pimecrolimus or ribavirin. The protein levels of DENV E were determined via western blotting. The expression of GAPDH was used as a loading control. (**c**) Quantification of the DENV E fold-change in protein levels relative to the DENV2 and vehicle-treated group. Data are expressed as mean ± SEM (n = 3 independent biological experiments, each conducted with triplicate technical repeats; ns, not significant; ***p < 0.001; comparisons made against the DENV2-infected and vehicle-treated group).

DENV2 infection leads to an increase in the number of cells expressing the E protein. However, we detected a significant inhibition of DENV envelope protein expression when using an immunofluorescence assay in infected Vero cells after treatment with pimecrolimus. We observed that treatment with 10–20 μM of pimecrolimus significantly reduced the intracellular expression of E protein (Fig. 4a). Similarly, pimecrolimus decreased the cellular expression of E protein in a dose-dependent manner (Fig. 4b,c).

### Pimecrolimus inhibits DENV2 replication and DENV2-induced focal necrosis in vivo

**Figure 5:**
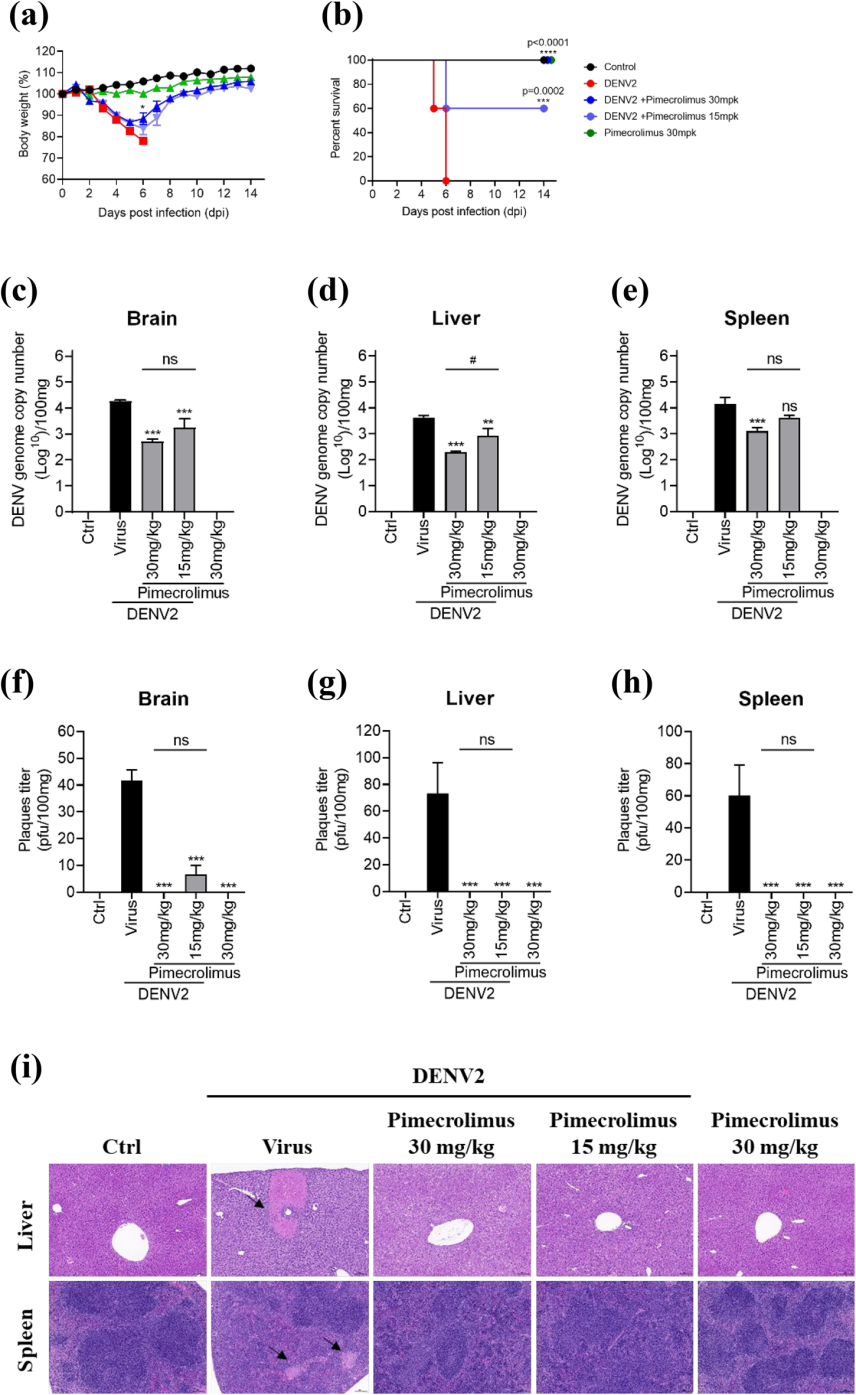
Antiviral effect of pimecrolimus against DENV2 in IFNAR1-transgenic mice. Six-week-old IFNAR1-transgenic mice were intraperitoneally inoculated with DENV2 (1.05 × 10^6^ PFU/mouse). Pimecrolimus was orally administered in mice (15 or 30 mg/kg) twice daily for 14 days. (**a**) Graph depicting changes in the body weight of mice across the different treatment groups, monitored for 14 dpi. (**b**) Survival rate of mice infected with DENV2 and treated with the indicated pimecrolimus concentrations. The DENV2 genome copy number was estimated in the supernatants of (**c**) brain, (**d**) liver, and (**e**) spleen tissue homogenates at 6 dpi, between the various treatment groups. Accordingly, the DENV2 plaque titer was analyzed in the supernatants of (**f**) brain, (**g**) liver, and (**h**) spleen tissue homogenates at 6 dpi, between the various treatment groups. (**i**) Representative hematoxylin and eosin staining images of the murine liver and spleen tissues. Black arrows indicate focal necrosis. Scale bar: 100 μm. All data are expressed as mean ± SEM (n = 3 independent biological experiments, each conducted with triplicate technical repeats; ns, not significant; *p < 0.05; ***p < 0.001; ****p < 0.0001; all comparisons were made against the DENV2-infected group).

Following up on our in vitro observations, we next assessed the potential antiviral efficacy of pimecrolimus in DENV2-infected interferon-alpha/beta receptor 1 (IFNAR1)-knockout mice. We observed no apparent differences between the control and the 30 mg/kg pimecrolimus only-treated groups. In contrast, DENV2-infected mice showed a decrease in body weight of more than 20% 6 days post-infection (dpi); notably, administration of 15 and 30 mg/kg of pimecrolimus to these mice resulted in a body weight decrease of only approximately 17% and 12%, respectively. Interestingly, their body weight was restored at 14 dpi (Fig. 5a). The DENV2-infected mice died within 6 dpi, whereas those treated with 15 and 30 mg/kg pimecrolimus significantly survived until 14 dpi (Fig. 5b).

The DENV2 infection resulted in a significant increase in the viral genome copy number in the brain, liver, and spleen compared to that in non-infected mice; however, pimecrolimus treatment significantly reduced the viral copy number in the brain and liver compared to that in the DENV2-infected group. In the spleen, treatment with 15 mg/kg pimecrolimus did not result in a marked reduction, whereas 30 mg/kg pimecrolimus significantly reduced DENV2 genome copy number (Fig. 5c–e). In addition, plaque assay analysis showed that pimecrolimus significantly reduced DENV2 production in the brain, liver, and spleen of mice (Fig. 5f–h).

We also examined whether pimecrolimus treatment inhibited DENV2 infection in mice. Our results showed that the brains of DENV2-infected mice were indistinguishable from those of control mice (data not shown). Interestingly, focal necrosis was observed in the spleen following DENV2 infection. The DENV2-induced focal necrosis in the liver and spleen was ameliorated by treatment with 15 or 30 mg/kg pimecrolimus (Fig. 5i). Taken together, these results highlighted that pimecrolimus was not cytotoxic at the concentrations used for our animal study and was effective as a protective antiviral agent against DENV2 infection in vivo.

## Discussion

DENV infection may lead to severe consequences, such as febrile illness, dengue fever, dengue hemorrhagic fever, and dengue shock syndrome^15^. DENV-infected mosquitoes, acting as vectors for virus transmission, also increase the risk of DENV infection. In this study, we used Vero and BHK-21 cells as they are susceptible to DENV infection. In previous in vitro studies, various host cells, including BHK-2^16^, Vero^17^, Vero E6^18^, Huh-7^19^, and A549^14^ cells, have been used for DENV infection. The antiviral agent ribavirin is currently used as the preferred treatment against infection by DENV^20,21^, ZIKV^21,22^, and hepatitis C virus (HCV)^23^. However, ribavirin fails to inhibit DENV infection *in vivo*^24^. In recent decades, many antiviral candidates for DENV infection have been identified; however, only a few have been further evaluated in preclinical or clinical trials, primarily because of issues with drug properties, including efficacy, specificity, toxicity, and stability^25^. Based on the findings of our current study, we suggest that pimecrolimus may be a potent novel antiviral agent against DENV2.

Pimecrolimus is effective in the treatment of inflammatory skin diseases. The antiviral activity of pimecrolimus against RNA viruses was first reported in an in vitro study using an antiviral agent against severe acute respiratory syndrome coronavirus 2 (SARS-CoV-2)^26^. In this study, we observed that pimecrolimus was efficient in attenuating and protecting against DENV2 and ZIKV infection. Zhou et al. have reported that ascomycin can inhibit the infection of Asian ZIKV strain SZ01^1^. However, in our study, we failed to observe the antiviral activity of ascomycin against African ZIKV MR766 strain (Supplementary Fig. S2).

Our findings indicate that pimecrolimus potently inhibits DENV2 infection at the early stages (0–2 h; Fig. 2b) and inhibits viral internalization (Fig. 2d). Considering that viral entry in host cells occurs within 2 h of adsorption^27^, the antiviral effects of pimecrolimus seem to be mostly associated with the inhibition of the DENV2 internalization process.

DENV does not replicate in wild-type mice because of its inability to antagonize the type I IFN signaling response. In contrast, IFNAR1^-/-^ mice are highly susceptible to DENV infection. DENV infection of IFNAR1^-/-^ mice resulted in the replication of DENV in serum and organs (liver and spleen)^28^. Furthermore, we demonstrated the antiviral effects of 15 and 30 mg/kg pimecrolimus doses against DENV2 in vivo. DENV2 infection in mice induces focal necrosis in the liver^29,30^. Interestingly, in our study in DENV2-infected mice, the spleen showed symptoms of focal necrosis in response to dengue infection (Fig. 5i).

In summary, our results suggest that pimecrolimus is a potential therapeutic agent against DENV2 infection; however, further investigation into delineating the full range of antiviral effects and the underlying mechanism of action of pimecrolimus against other DENV2 serotypes is warranted.

## Methods

### Ethics statement

All experiments were performed in accordance with relevant guidelines and regulations. All animal experiments were carried out in strict accordance with the Animal Research: Reporting of In Vivo Experiments (ARRIVE; http://arriveguidelines.org) guidelines and were approved by the Institutional Animal Care and Use Committee (IACUC; approval no. KDCA-IACUC-23–021) at the Korea Disease Control and Prevention Agency. Mice were housed in BLS mouse cages (INNOVIVE, CA, USA) with individually ventilated racks with four mice per cage. The experimental animal room was maintained within set parameters: 21–24 °C, 50–70% humidity, and a 12:12 h light:dark cycle. The humane endpoint in this study was a body weight decrease of > 20%. All mice were sacrificed using tiletamine and zolazepam (Zoletil, 30 mg/kg), xylazine (Rumpun, 10 mg/kg), and CO_2_ inhalation.

### Cells, viruses, and reagents

Vero and BHK-21 cells (ATCC, Manassas, VA, USA) were maintained in minimum essential Medium (MEM) (GenDEPOT, TX, USA). Vero E6 cells (ATCC) were cultured in Dulbecco’s modified Eagle’s medium (Gibco Laboratories, NY, USA), and Huh-7 cells (KCLB, Seoul, Korea) were maintained in RPMI 1640 medium (Gibco Laboratories). The media were supplemented with 10% fetal bovine serum (FBS), 1 unit/mL of penicillin, and 1 μg/mL of streptomycin (Gibco Laboratories). Cells were cultured in 37 ℃ incubator (Eppendorf, Hamburg, Germany) with 5% CO_2_. DENV2 (ATCC) and ZIKV strain MR 766 (ATCC) were maintained by incubation of cells at 37 ℃ and 5% CO_2_. Pimecrolimus, ascomycin, and tacrolimus were purchased from Applied Biosystems (Foster City, CA, USA). Ribavirin was purchased from Sigma-Aldrich (St. Louis, MO, USA).

### Cell viability assay

Vero and BHK-21 cells (3 × 10^4^ cells/well) were seeded in 96-well culture plates (Corning, NY, USA). After 24 h, Vero and BHK-21 cells infected with DENV2 were incubated for an additional 96 h and 48 h, respectively. The DENV2 infection medium was then replaced with MEM supplemented with 2% FBS. Cell cytotoxicity was measured using the WST-1 assay (EZ-Cytox cell viability assay kit, Dogen bio, Seoul, Korea). EZ-Cytox cell viability assay kit reagents were added to the cells and incubated for a further 4 h at 37 ℃ and 5% CO_2_, according to the manufacturer’s protocol. The absorbance at 450 nm was measured using a microplate reader. Images of crystal violet-stained cells were obtained using an S6 CORE analyzer (C.T.L., OH, USA).

### Real-time PCR

Total RNA was isolated from cells using a QIAamp viral RNA Mini Prep kit (Qiagen, Valencia, CA, USA) according to the manufacturer’s protocol. Total RNA concentration was measured using the Nanodrop One^©^ (Thermo Scientific, MA, USA). Real-time PCR amplification of DENV2 was performed using the PowerCheck DENV Serotype Real-time PCR Kit (Kogen Biotech, Seoul, Korea). The PCR conditions were as follows: 45 °C for 30 min, 95 °C for 10 min, followed by 40 cycles of 95 °C for 45 s and 60 °C for 30 s. Relative mRNA levels for each reaction were normalized to those of *β-actin* using the Power SYBR™ Green RNA-to CT™ 1-Step Kit (Thermo Scientific). We used the following primers: *β-actin*-sense, 5’-AAGGATTCATATGTGGGCGATG-3’ and *β-actin*-antisense, 5’-TCTCCATGTCGTCCCAGTTGG-3. ’ Real-time PCR was performed at a temperature cycling of 95 ℃ for 10 min (1 cycle), followed by 40 cycles of 95 ℃ for 10 s and 60 ℃ for 30 s.

### Western blotting

The protein concentration of lysed cell extracts in RIPA buffer (Sigma-Aldrich) was determined using a Pierce™ BCA Protein Assay Kit (Thermo Scientific), and samples of equal protein amounts (20 µg) were loaded onto 4–12% gradient SDS-PAGE gels and subsequently transferred to a PVDF membrane. The membranes were blocked with 5% skim milk in TBS with 0.5% Tween 20 (TBST). The membranes were subsequently incubated with anti-DENV E protein (GeneTex, Carlsbad, CA, USA) and anti-GAPDH antibody (Thermo Scientific) overnight at 4 ℃. After extensive washing in TBST, the immunoblots were probed with secondary anti-rabbit and anti-mouse IgG and HRP-linked antibodies (Cell Signaling, Danvers, MA, USA) for 1 h at room temperature. The SuperSignal® West Dura Extended Duration Substrate (Thermo Scientific) was used to produce the chemiluminescent signal on the immunoblots. Images of the blots were acquired using the AMERSHAM ImageQuant 800 western blot imaging system (Cytiva, Marlborough, MA, USA). Band fluorescence intensity was analyzed using ImageJ version 1.53 k software (https://imagej.nih.gov/ij) (NIH, Bethesda, MD, USA).

### Immunofluorescence

Vero cells (2 × 10^5^ cells/well) were seeded in four-well culture plates (Nalge Nunc International, Rochester, NY, USA), with coverslips placed on each well. After 24 h, cells were infected with 0.1 MOI of DENV2 at different concentrations and incubated at 37 ℃ and 5% CO_2_ for 48 h. The cells that adhered to the coverslips were washed with PBS, fixed with 4% formaldehyde for 20 min, and blocked with 5% skim milk. Cells were stained with anti-DENV E protein antibody (1:500 dilution; GeneTex) and incubated for 1 h at room temperature. After washing with PBS, the cells were incubated with Alexa Fluor 488 goat anti-mouse IgG (1:500 dilution; Invitrogen, Carlsbad, CA, USA) for 4 h at room temperature. After washing with PBS, coverslips were stained with 4’,6-diamidine-2-phenylindole (DAPI). Slides were visualized using an FV1000 confocal microscope (Olympus, Tokyo, Japan).

### Time-of-addition assay

An addition assay was performed to evaluate the effects of pimecrolimus on the DENV life cycle. Vero cells (3 × 10^4^ cells/well) were seeded in 96-well culture plates. After 24 h, the cells were infected with 0.1 MOI of DENV2 for 1 h and then washed with PBS. Virus infection medium was then added to the cells. Pimecrolimus at a concentration of 20 μΜ was then added to cells at eight different time points after infection (0, 1, 2, 4, 6, 8, 10, and 12 h). After 12 h, the total RNA was extracted using a QIAamp® Viral RNA mini kit, and the normalized DENV2 genome expression was analyzed using real-time PCR.

### Viral binding and internalization assays

Viral binding and internalization assays were performed as previously described^31^, with minor modifications. For the viral binding assay, Vero cells (2.5 × 10^4^ cells/well) were seeded in 96-well culture plates. After 24 h, the cells were infected with 0.1 MOI of DENV2, treated with the indicated concentrations of pimecrolimus, and incubated for 1 h at 4 °C. The cells were then washed twice with ice-cold PBS, and the medium was replaced with MEM supplemented with 2% FBS. Following incubation at 37 °C under 5% CO_2_ for 96 h, the cell viability assay was performed. The DNEV2 genome copy number in Vero cells was measured at five different time points after infection (1.5, 3, 6, 12, and 24 h) using real-time PCR.

For the viral internalization assay, Vero cells were infected with 0.1 MOI of DENV2 for 1 h at 4 °C. The infected cells were washed twice with ice-cold PBS, treated with different concentrations of pimecrolimus in MEM supplemented with 2% FBS, and incubated for 1 h at 37 °C. The cells were then washed twice with PBS and treated with citrate buffer (40 mM citric acid, 10 mM potassium chloride, and 135 mM sodium chloride, pH 3) for 1 min. After washing, the cells were incubated with MEM supplemented with 2% FBS at 37 °C under 5% CO_2_ for 96 h. Cell cytotoxicity was measured using the cell viability assay. The DNEV2 genome copy number in Vero cells was measured at five different time points after infection (1.5, 3, 6, 12, and 24 h) using real-time PCR. The serum of DENV2-infected mice was used as control in both assays.

### Time-based growth inhibition using real-time PCR and plaque assay

Vero cells (3 × 10^4^ cells/well) were seeded in 96-well culture plates for 24 h, after which the cells were infected with 0.1 MOI of DENV2 for 1 h and washed with PBS. After 1 h, pimecrolimus (20 μΜ), ascomycin (20 μΜ), tacrolimus (20 μΜ), or ribavirin (500 μΜ) was added to the cell culture. Vero cells were harvested at the indicated time points (3, 6, 9, 12, 24, 48 and 72 hpi). Total RNA was isolated from the cells at the indicated time points, and normalized DENV2 genome expression was analyzed using real-time PCR. The inhibition of plaque formation at different time points was assessed using a plaque assay.

### Plaque reduction assay

BHK-21, Vero E6, or Huh-7 cells were seeded at 3 × 10^5^ cells/well in six-well culture plates (Corning). After 24 h, 50 plaque-forming units (PFU) of DENV2-infected BHK-21, Vero E6, or Huh-7 cells and 50 PFU of ZIKV-infected Vero cells were incubated at 37 ℃ and 5% CO_2_ for 1 h. The culture medium was then replaced with MEM containing 2% FBS and 0.5% LMP agar (Invitrogen) supplemented with different concentrations of compounds in each experiment. After 2 days (for ZIKV-infected Vero cells), 4 days (for DENV2-infected BHK-21 cells), or 7 days (for DENV2-infected Vero E6 and Huh7 cells), cells were fixed and stained with 4% paraformaldehyde, 0.2% crystal violet (Sigma-Aldrich), and 20% EtOH for 4 h. The viral titer was expressed as PFU/mL.

### Mice and virus infection

For this study, 6-week-old female B6(Cg)-Ifnar1tm1.2Ees/J mice (MMRRC Strain #028288-JAX) were purchased from the Jackson Laboratory (Bar Harbor, ME, USA). The mice (n = 8 per group) were acclimated for 7 days at the ABL2 facility of the Korea Disease Control and Prevention Agency. For antiviral activity experiments, mice were infected intraperitoneally with DENV2 (1.05 × 10^6^ PFU/mouse). Pimecrolimus was administered orally, twice daily at 15 and/or 30 mg/kg after DENV2 infection. The body weight and survival rate of the mice were monitored for 14 days, and the viral genome copy number and pathological symptoms were measured at 6 dpi. Mice were euthanized at 6 dpi to collect the brain, liver, and spleen, which were analyzed using real-time PCR.

### Histology

The livers and spleens of mice were fixed with 4% formaldehyde for 24 h, then dehydrated in serial gradients of ethanol, washed in xylene, and embedded in paraffin. The tissue was then sliced into 5-μm-thick sections and stained with hematoxylin and eosin. Liver necrosis was assessed using a Panoramic Scan II slide scanner (3D Histech, Budapest, Hungary).

### Statistical analysis

Statistical analyses were conducted using one-way analysis of variance followed by the Newman–Keuls multiple comparison test. The survival rate of the animals was estimated using the log-rank (Mantel–Cox) test. All statistical analyses were performed using the GraphPad Prism version 9 (https://www.graphpad.com) (GraphPad Software, La Jolla, CA, USA).

### Supplementary Information

Supplementary Figures.

## Data Availability

The data accompanying this study will be made available from the corresponding author on reasonable request.
