# Supplementary material for: Antiviral activity of pimecrolimus against dengue virus type 2 infection in vitro and in vivo
Source: Sci Rep. 2024 Jun 10;14:13303. doi: 10.1038/s41598-024-61127-x (PMC11164929; doi:10.1038/s41598-024-61127-x)
Supplement: Supplementary file 1 — Supplementary Figures. [file 41598_2024_61127_MOESM1_ESM.docx]

**Supplementary Information**

**Antiviral activity of pimecrolimus against dengue virus type 2 infection *in vitro* and *in vivo***

Seong-Ryeol Kim ^a, 1^, Jung-Min Lee ^a, 1^, Hae Ji kang ^a^, Jungsang Ryou ^a, *^, and Sang-Mu Shim ^a, *^

^a^Division of Acute Viral Diseases, Centers for Emerging Virus Research, National Institute of Infectious Disease, Korea National Institute of Health, Korea Disease Control and Prevention Agency, Cheongju-si, Chungcheongbuk-do, Republic of Korea

* Corresponding author

^1^ These authors contributed equally to this paper

Author e-mail addresses: Seong-Ryeol Kim (ksr12134@korea.kr), Jung-Min Lee ([wjdalslee@korea.kr](mailto:wjdalslee@korea.kr)), Hae Ji kang ([haeji.kang@korea.kr](mailto:haeji.kang@korea.kr)), Jungsang Ryou ([zenith@nih.go.kr](mailto:zenith@nih.go.kr)) and Sang-Mu Shim ([smshim@korea.kr](mailto:smshim@korea.kr))

**
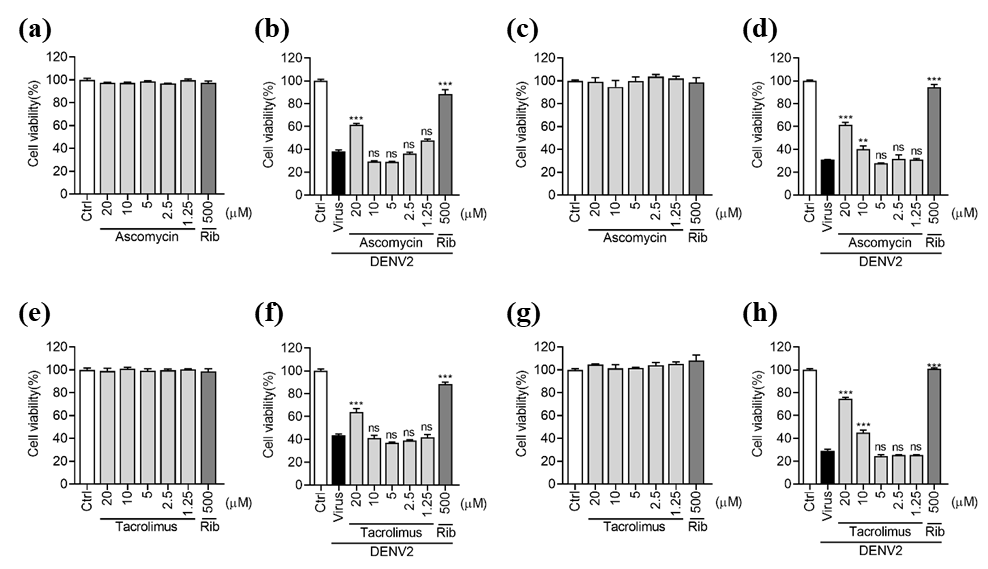
**

**Supplementary Figure S1. Ascomycin and tacrolimus exhibit antiviral activity against DENV2 infection**

(**a**, **b**) Vero cells were treated with the indicated concentrations of ascomycin or ribavirin (**a**) in the absence of virus, or (**b**) infected with 0.1 multiplicity of infection (MOI) of DENV2, for 4 dpi. (**c**, **d**) BHK-21 cells were treated with the indicated concentrations of ascomycin or ribavirin (**c**) in the absence of virus, or (**d**) infected with 0.1 MOI of DENV2, for 2 dpi. (**e**, **f**) Vero cells were treated with indicated concentrations of tacrolimus or ribavirin (**e**) in the absence of virus, or (**f**) infected with 0.1 MOI of DENV2, for 4 dpi. (**g**, **h**) BHK-21 cells were treated with the indicated concentrations of tacrolimus or ribavirin (**g**) in the absence of virus, or (**h**) infected with 0.1 MOI of DENV2, for 2 dpi. Cell viability was evaluated using an EZ-Cytox cell viability assay. Data are expressed as mean ± SEM (n=3 independent biological experiments, each conducted with triplicate technical repeats; ns, not significant; ***p*<0.01; ****p*<0.001; comparisons made against the DENV2-infected group).

**
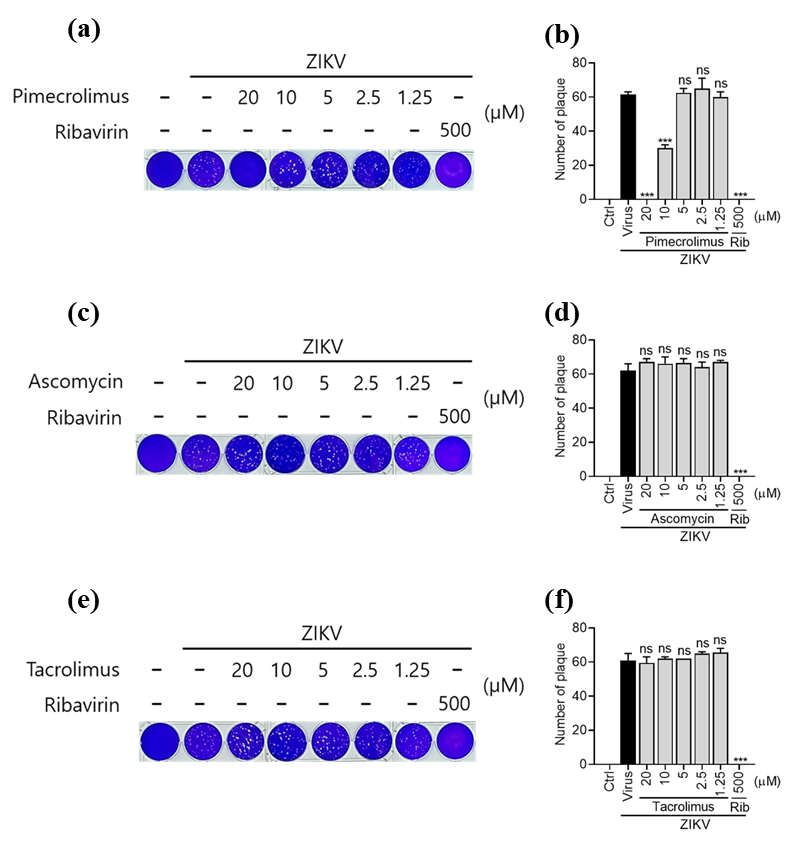
**

**Supplementary Figure S2.** **Pimecrolimus exhibits antiviral activity against ZIKV infection**

Antiviral effects of analog compounds against ZIKV assessed via plaque reduction assays. (**a**) Representative crystal violet staining images of ZIKV-infected Vero cells treated with the indicated concentrations of pimecrolimus or ribavirin. (**b**) Comparison of the relative number of plaques formed across the different pimecrolimus concentrations. (**c**) Representative crystal violet staining images of ZIKV-infected Vero cells treated with the indicated concentrations of ascomycin or ribavirin. (**d**) Comparison of the relative number of plaques formed across the different ascomycin concentrations. (**e**) Representative crystal violet staining images of ZIKV-infected Vero cells with the indicated concentrations of tacrolimus or ribavirin. (**f**) Comparison of the relative number of plaques formed across the different tacrolimus concentrations. Data are expressed as mean ± SEM (n=3 independent biological experiments, each conducted with triplicate technical repeats; ns, not significant; ****p*<0.001; comparisons made against the DENV2-infected group in each graph).
